# A Mathematical Model of Chikungunya Dynamics and Control: The Major Epidemic on Réunion Island

**DOI:** 10.1371/journal.pone.0057448

**Published:** 2013-03-06

**Authors:** Laith Yakob, Archie C. A. Clements

**Affiliations:** School of Population Health, University of Queensland, Brisbane, Queensland, Australia; Massey University, New Zealand

## Abstract

Chikungunya is a re-emerging arboviral disease transmitted by *Aedes* spp. mosquitoes. Although principally endemic to Africa and Asia, recent outbreaks have occurred in Europe following introductions by returning travellers. A particularly large outbreak occurred on Réunion Island in 2006, the published data from which forms the basis of the current study. A simple, deterministic mathematical model of the transmission of the virus between humans and mosquitoes was constructed and parameterised with the up-to-date literature on infection biology. The model is fitted to the large Réunion epidemic, resulting in an estimate of 4.1 for the type reproduction number of chikungunya. Although simplistic, the model provided a close approximation of both the peak incidence of the outbreak and the final epidemic size. Sensitivity analysis using Monte Carlo simulation demonstrated the strong influence that both the latent period of infection in humans and the pre-patent period have on these two epidemiological outcomes. We show why separating these variables, which are epidemiologically distinct in chikungunya infections, is not only necessary for accurate model fitting but also important in informing control.

## Introduction

Chikungunya is an alphavirus that infects humans through bites from *Aedes* spp. mosquitoes. Symptoms are similar to those of dengue fever during the acute phase and include rash and high fever that, in a small proportion of cases, can develop into a life-threatening haemorrhagic fever [1]. Additionally, joint pain that is frequently associated with infection can persist for over a year [2], and is responsible for its name which means “that which bends” in the Makonde language of Southern Tanzania and Northern Mozambique. In 2004, a major epidemic in Lamu, Kenya resulted in 13,500 cases [3]. This epidemic sparked a four-year period in which the virus spread through numerous islands of the Indian Ocean, India and parts of Southeast Asia [4]. Cases were imported to Europe and North America through returning travellers, and subsequent autochthonous transmission events occurred due to the wide geographical distribution of the vectors [4].

The French island of Réunion in the Indian Ocean experienced a major outbreak where, during 2005–6, approximately 266,000 of the 785,000 inhabitants were infected, causing or contributing to over 200 deaths [5], [6]. Following the international WHO alert in March 2005, an island-wide operational surveillance system for chikungunya infections was set up to characterise cases and to monitor trends. However, by December 2005, the numbers of cases exceeded the capacity of the surveillance system and incidence was extrapolated from a sentinel network of physicians, and later confirmed through a combination of hospital activity data, self-reporting by the population and seroprevalence data [5]. Data from Renault et al. (2007) forms the basis of the current epidemiological study.

We constructed an ordinary differential equation model to simulate the transmission of infection between humans and *Aedes albopictus* – the principle vector on Réunion during the major epidemic [6]. The ranges for the biological components of the model were provided by a review of the literature. The model was then used to calculate the basic reproduction number (and type reproduction number) of chikungunya by fitting it to the Réunion data. Monte Carlo analysis was performed to determine the sensitivity of infection dynamics to the parameters. Accurate estimates of the type reproduction number [7] and model sensitivity to its biological components is critical to informing control and, following the results, we discuss how our study contributes to the limited intervention strategies available for this disease.

## Methods

**Figure 1:**
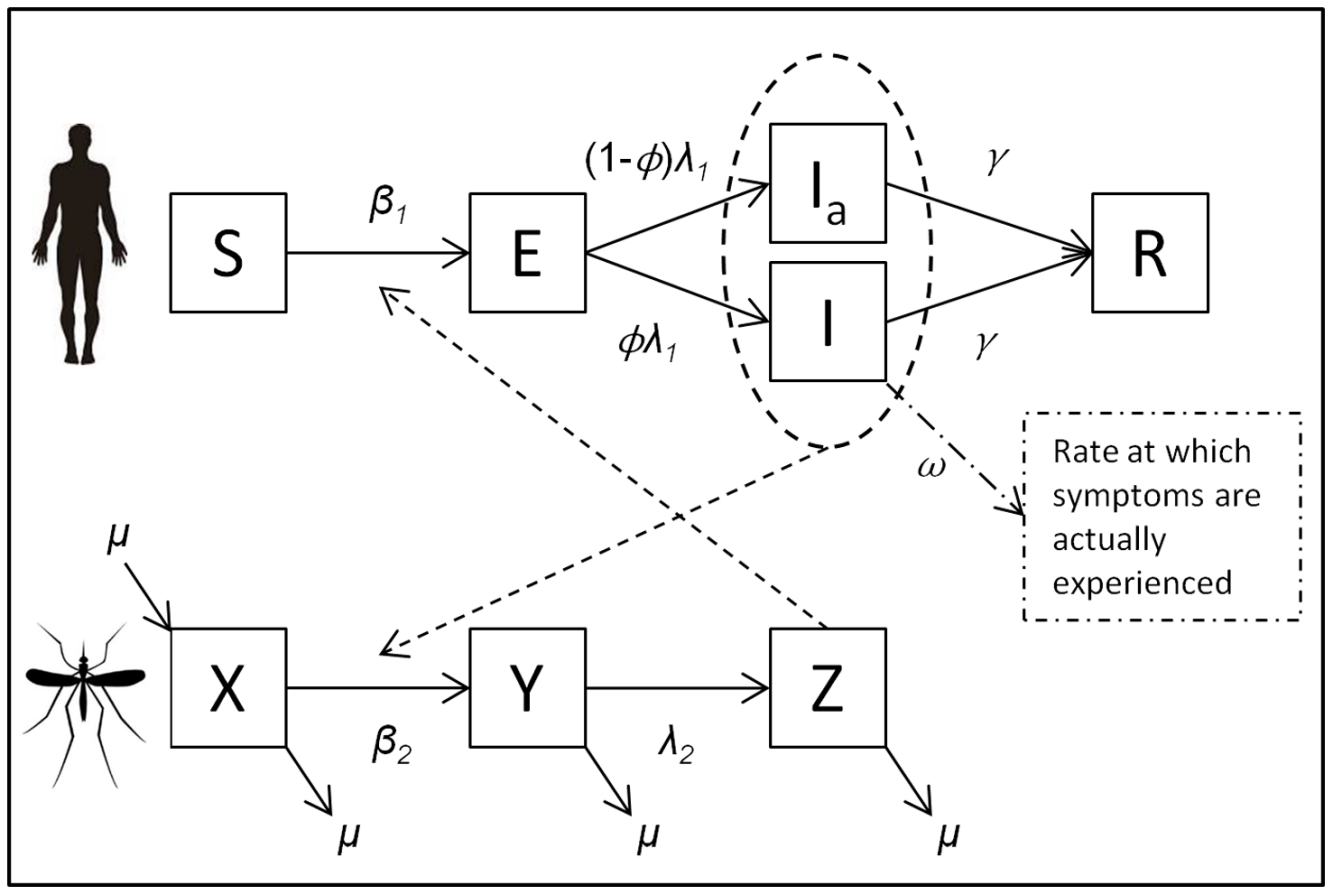
Compartmental construction of the epidemiological model for Chikungunya transmission. Susceptible humans (S) are exposed to infection (E) before becoming infectious (I_a_ asymptomatically, or I symptomatically) and then recover (R). Susceptible mosquitoes (X) are exposed to infection (Y) before becoming infectious (Z). Transmission from mosquito-to-human and vice versa is denoted by the broken lines indicating a mosquito bite. Rates of change between compartments are denoted by corresponding Greek letters.

Figure 1 describes the compartmental design of the model. The proportion of susceptible people (S) is exposed to the pathogen (E) when bitten by an infectious mosquito. Following the latent period of infection, people become either symptomatically Infectious (I) or asymptomatically Infectious (I_a_) before recovering (R). Similarly, following an infectious bite, the proportion of susceptible mosquitoes (X) becomes exposed to the pathogen (Y) before themselves becoming infectious (Z). The corresponding equations that describe the infection dynamics are:
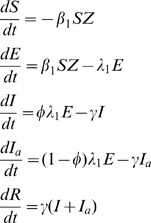


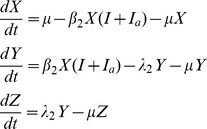



*β_1_* is the rate at which mosquitoes infect humans (Table 1 describes model parameters and variables). In this way, *β_1_* is the equivalent to *abm* in Macdonald’s model (where *a* is the bite rate, *b* is the parasite transmissibility to humans and *m* is the ratio of mosquitoes to humans) [8]. Following convention of this model and its modern-day adaptations [9], the rate at which mosquitoes infect humans is dependent on the ratio of mosquitoes to humans but the rate at which humans infect mosquitoes, *β_2_*, is independent of this ratio. ϕ is the proportion of infected people who develop symptoms, in the range 0.83–0.97 [2], [5], [10]. *λ_1_* is the inverse of the latent period of infection, which is estimated to be between 2 and 6 days [10]. *γ* is the rate of recovery, which is assumed to take between 1 and 7 days [11]. For chikungunya, the latent period is distinct from the pre-patent period, *ω* ^−1^, which typically lasts between 4 and 7 days [12]. The latent period is the number of days before an individual becomes infectious and the pre-patent period is the number of days before a patient exhibits symptoms. Importantly, because case notification was based on the reporting of symptoms, it is the symptomatically *infected*, and not necessarily just the symptomatically *infectious*, that is relevant to the fitted data.

**Table 1:** The parameters and variables (with units) of the Chikungunya model.

Symbol	Definition (units)
***S***	Susceptible hosts (proportion)
***E***	Exposed hosts (proportion)
***I***	Symptomatically infectious hosts (proportion)
***I_a_***	Asymptomatically infectious hosts (proportion)
***R***	Recovered hosts (proportion)
***X***	Susceptible mosquitoes (proportion)
***Y***	Exposed mosquitoes (proportion)
***Z***	Infectious mosquitoes (proportion)
***β_1_***	Mosquito-to-human transmission (number of mosquito bites per human per day allowing for imperfect pathogen transmission)
***β_2_***	Human-to-mosquito transmission (per day bite rate also allowing for imperfect pathogen transmission)
ϕ	Hosts that develop symptoms (proportion)
***1/λ_1_***	Host latent period (from ‘infected’ to ‘infectious’, days)
***1/λ_2_***	Mosquito latent period (from ‘infected’ to ‘infectious’, days)
***γ***	Host recovery rate (per day)
***1/ω***	Host pre-patent period (from ‘infected’ to symptoms development, days)
***1/μ***	Mosquito life span (days)

SIR-type models describe infection prevalence (not incidence). In order to compare weekly incidence data with our model output, new infections were tracked each day. The symptomatic proportion (ϕ) of these new infections from *ω* ^−1^ days ago thereby represents the current day’s incidence of symptomatic infection. This daily symptomatic incidence was summed every 7 days and then compared with (and fitted to) the weekly incidence data collected by Renault et al. (2007).

Parameters describing mosquito biology and infection include *λ_2_*, the latent period of infection in mosquitoes, which is between 2 and 6 days for *A. albopictus* [13] and, *μ*, the mortality rate of the mosquitoes which is inverse of the average life expectancy of 20–30 days [14]. Mosquito births are set to balance deaths. Hence, a stable mosquito population is assumed for this tropical island which experiences temperature and rainfall conditions suitable for year-round *A. albopictus* breeding [15]. If new empirical evidence demonstrated marked seasonal variation in mosquito populations, our simple framework could easily be adapted to include a sinusoidal seasonal forcing function in mosquito dynamics, as with the study of Bacaër [16].

Next generation matrix methods were used to calculate the basic reproduction number, *R_0_* [17]. The transmission matrix, *T*, denotes the pathogen passing between all stages of the infection subsystem:
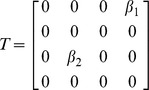



The transition matrix, *Σ*, denotes all other transitions to and from the infection subsystem:
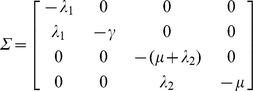



The model was fitted to the incidence data using the least squares method whereby parameters were allowed to vary within the range described by the clinical and entomological literature. Where no values were obtainable from the literature, i.e. for the transmission coefficients, *β_1_* and *β_2_*, triangular probability distributions were generated whereby the modal value was the best-fit parameterisation generated through least squares, the minimum value was set to 50% of the least-squares estimate and the maximum value was set to 150% of the least-squares estimate. 10,000 runs of a Monte Carlo simulation were performed using these best-fit values and distributions, and the means (and standard deviations) for the incidence during the epidemic’s peak and final epidemic size were calculated. Sensitivities of these two model outputs to all model inputs were then estimated by allowing each parameter to vary by ±10% for 10,000 runs of a Monte Carlo simulation and calculating the Spearman’s rank correlation coefficients [18]. Knowledge of model sensitivity was then used to inform a suite of disease control scenarios.

## Results

**Figure 2:**
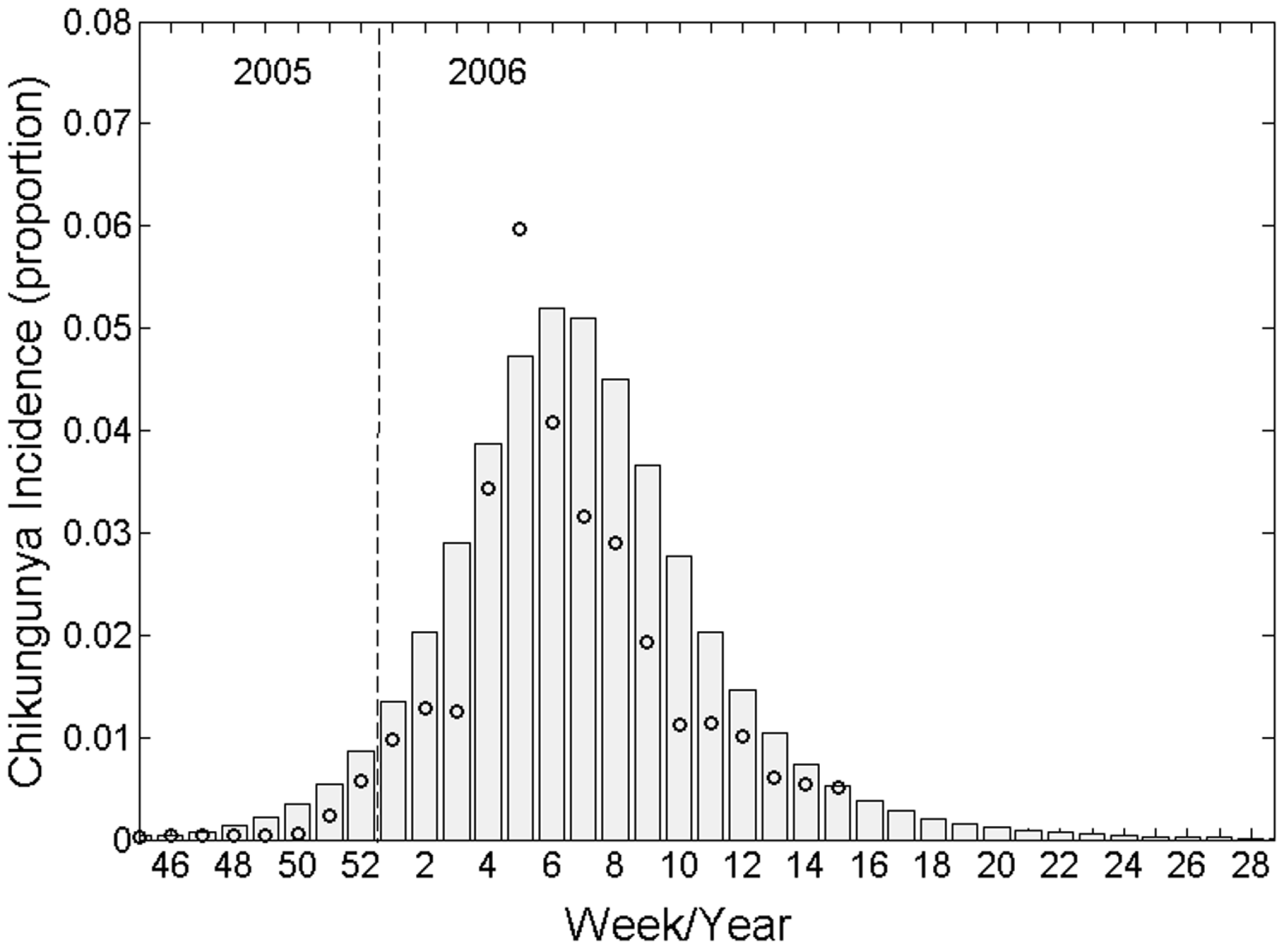
Mathematical model output (bars) fitted to weekly Chikungunya incidence data (circles) collected during the 2005–6 epidemic on Réunion island, Indian Ocean.

*R_0_* is the spectral radius of –*TΣ* ^−1^:
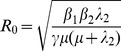



However, we are interested in the total number of secondary infections in humans originating from a human infection, not the average number of infections from human-to-mosquito and mosquito-to-human [7], [19]. In the terminology of Roberts and Heesterbeek (2003), this is the ‘type reproduction number’ and, in this case, is simply calculated as the *R_0_* squared (*R_T_* = *R_0_* ^2^). Obviously, both metrics have identical epidemic thresholds of 1. Weekly incidence from our best-fit model is plotted against the original data collected from Réunion Island in 2005–6 in Figure 2. We calculated the resulting *R_T_* value for the epidemic to be 4.1.

**Figure 3:**
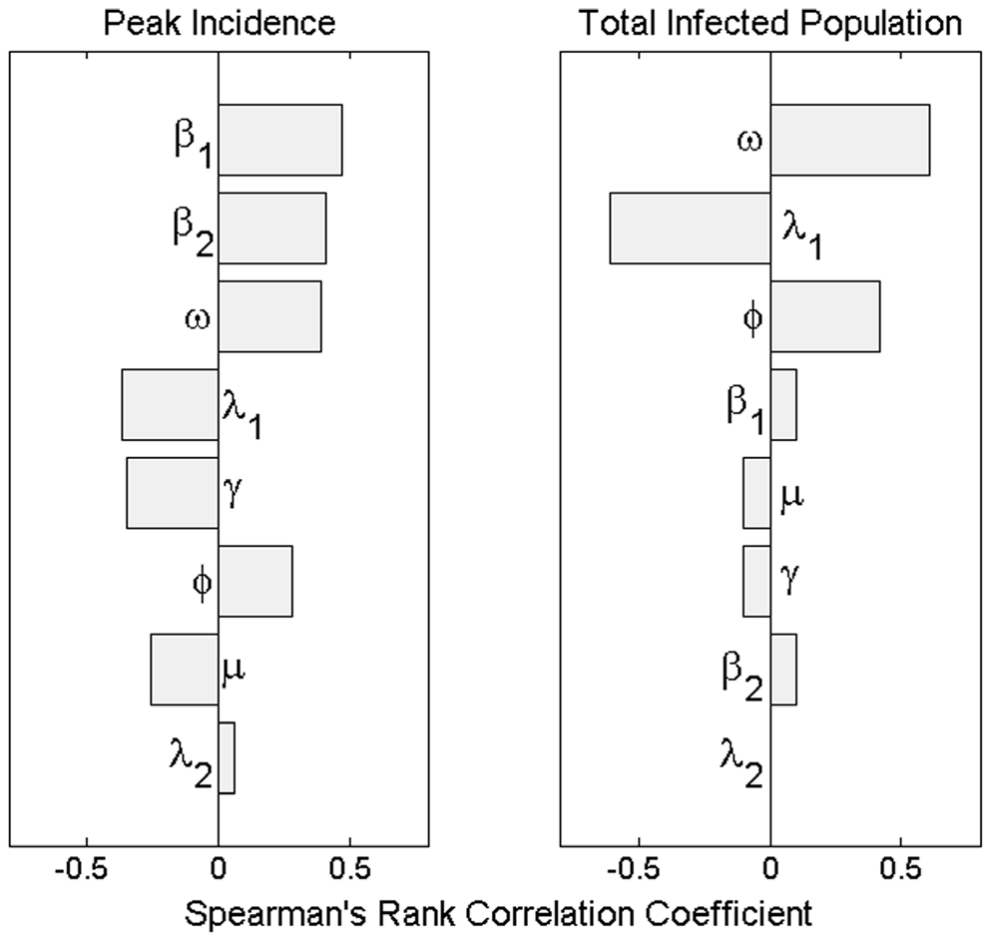
Spearman’s rank correlation coefficients demonstrating model output sensitivity to input parameters. 10,000 iterations of a Monte Carlo simulation were performed allowing each input parameter to vary by ±10% around its modal value.

Allowing the best-fit parameterisation to inform the modal *β_1_* and *β_2_* values of triangular probability distributions, we ran 10,000 Monte Carlo simulations and calculated the mean peak incidence to be 5.3% of the population of Réunion (standard deviation 1.8%), representing a close estimate of the ∼6% described in the data. The total infected population simulated by our model was 42.0% (standard deviation 9.0%) which compares well with the 35–38% estimated following the outbreak [5], [6]. These modal and best-fit values are as follows: *β_1_* = 0.14, *β_2_* = 0.40, γ = 0.25, *λ_1_* = 0.50, *λ_2_* = 0.50, ϕ = 0.97, *ω* = 0.25, *μ* = 0.05. Allowing all parameters to vary by ±10% for 10,000 Monte Carlo simulation runs, sensitivity of both model outputs (peak incidence and final epidemic size) to model input parameters is described by Spearman’s rank correlation coefficients and plotted in Figure 3.

Intuitively, peak incidence is most sensitive (and positively correlated) to the parameters determining the force of infection (*β_1_* and *β_2_*). This output was also sensitive to (and positively correlated with) the rate of symptoms onset (*ω*) and negatively correlated with the rate at which a human becomes infectious (*λ_1_*) and the rate at which humans recover from infection (*γ*). For the final epidemic size, parameters of greatest influence included the rate of symptoms onset (*ω*), the rate at which a human becomes infectious (*λ_1_*) and the proportion of infections that are symptomatic (ϕ). All other parameters were substantially less influential (Figure 3).

**Figure 4:**
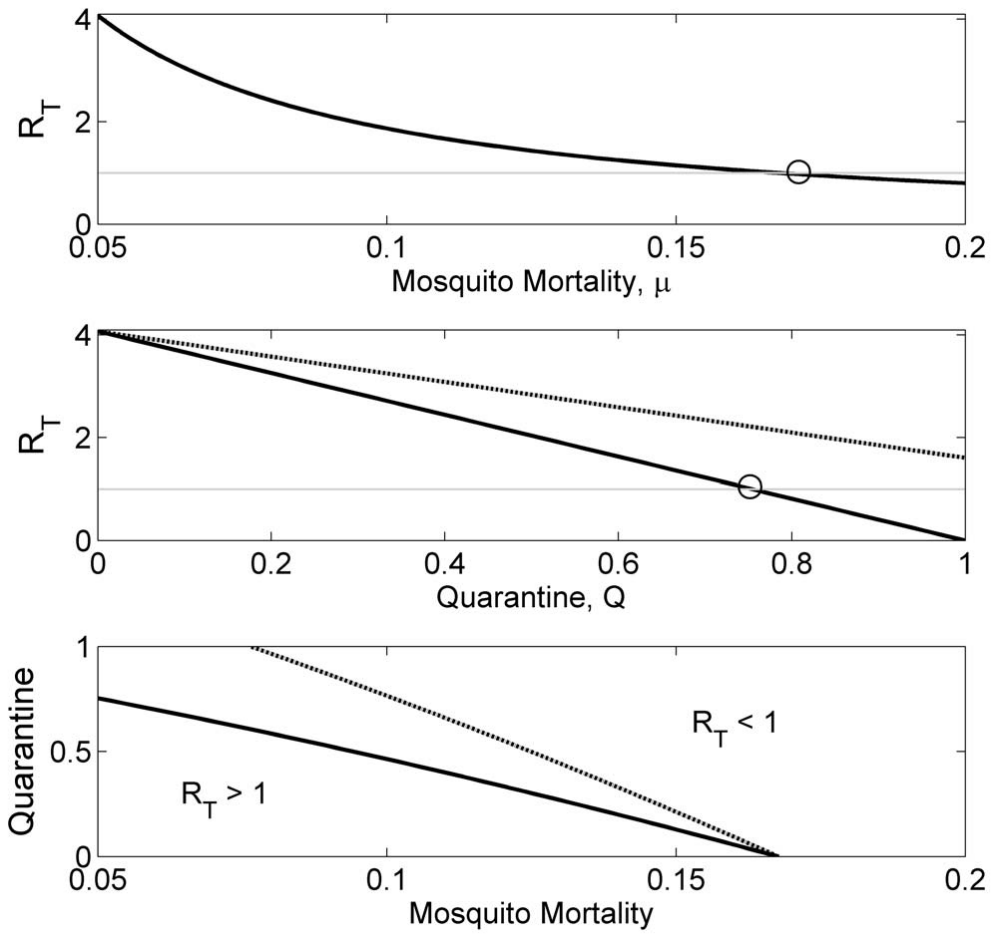
Controlling a chikungunya epidemic. Top, the reduction in the type reproduction number (*R_T_*) as a function of vector control (increased mosquito mortality rate). Middle, the reduction in the type reproduction number as a function of quarantine (solid line, *Q_1_*– pre-emptive isolation through screening or self-reporting mosquito bites, and, broken line, *Q_2_*– isolation following symptoms onset). Bottom, the combinations of vector control with quarantining (solid line *Q_1_* and broken line *Q_2_*) required to reduce the *R_T_* below unity.

Traditionally, adulticidal insecticides are employed to prevent, or curtail, vector-borne disease transmission. Following the chikungunya epidemic on Réunion, an island-wide mass-spraying effort was initiated [5]. Adulticides are modelled by increasing the mosquito mortality rate [9], [20]–[22]. The critical mortality rate (μ*) that must be achieved to prevent transmission is calculated as:
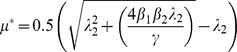



The lack of available treatment or vaccines for chikungunya limits the opportunities for reducing the transmission intensity. However, one potential control tool that has not been assessed for chikungunya (empirically or theoretically) is quarantining the infectious individuals. Sensitivity analysis demonstrated the influence that both the human latent and pre-patent periods have on transmission. For chikungunya, and any other disease whereby hosts are infectious before symptoms develop, there are different functional types of quarantine. ‘Type 1’ quarantine assumes knowledge of infection status without symptoms onset. This would be the case if an accurate, cheap and rapid screen became available to at-risk populations, or if people were isolated following self-reported mosquito bites. (This latter scenario represents quite an extreme level of cautiousness.) Type 1 quarantine threshold for eliminating transmission is calculated as:
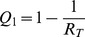



‘Type 2’ quarantine makes (the more realistic) assumption that isolation only occurs once symptoms have already developed. Type 2 quarantine threshold accounts for the additional delay between infectiousness and symptoms onset, and is calculated as:
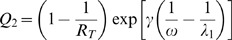



These threshold conditions for eliminating transmission are shown in isolation and in combination in Figure 4. The figure demonstrates the superiority of vector control when compared with quarantining. It also shows that the additional delay in isolating symptomatic, rather than bitten or screened, individuals (*Q_2_* vs *Q_1_*) can make the difference between an epidemic that is preventable and an epidemic that cannot be prevented through quarantining. Finally, it describes the reduced effort required of vector control in curtailing an epidemic as a function of the two different quarantine strategies.

## Discussion

Surprisingly, there have been relatively few mathematical models published on chikungunya transmission dynamics, and, to the best of our knowledge, the present study is the first to explore the sensitivity of chikungunya transmission to model input parameters. Bacaër [16] produced the first ordinary differential equation model and calculated the *R_T_* to be 3.4 after fitting it to the Réunion outbreak data. Dumont and Chiroleu [23] estimate *R_T_* between 1.46 and 1.78 for the same epidemic. However, in both analyses, the authors did not account for the fact that there is a time delay between becoming infectious (their model output) and the onset of clinical symptoms (the data). It also appears as though infection *prevalence* output from both models were fitted to the infection symptom *incidence* data. Massad et al. [24] parameterised their model based on the risk of an outbreak in Singapore and calculated an *R_T_* of 1.22. This very low estimate appears to be the result of an assumed short lifespan of the vector (10 days) combined with a very high extrinsic incubation period of 15 days [24]. More recently, Poletti et al. [25] described a vector-centric model of chikungunya, with parameterisation based on data from the 2007 Italian outbreak. Our calculation for *R_T_* falls in the middle of their estimated range of between 1.8 and 6. Using the Bayesian framework for analysing outbreak data developed by Cauchemez et al. (2006), Boelle et al. (2008) derived a value of 3.7 for the *R_T_* [26], [27]. Dengue, a highly related alphavirus also transmitted by *Aedes* spp. that has had considerably greater research effort than chikungunya, has comparable *R_T_* estimates of 4.3–5.8 [28], 2.7–11.6 [29] and 3.8–5.1 [30]. Note than in each of these previously published studies, the threshold is described as the basic reproduction number, but it is the type reproduction number that is actually presented. As described earlier, squaring the spectral radius of the next generation matrix does not affect the threshold (R_0_ = 1 ≡ R_T_ = 1). However, making the distinction becomes important when assessing control because R_0_ will always underestimate the level of control required for elimination of a vector-borne disease.

Our analysis has taken advantage of the recent surge in chikungunya research resulting from the spate of epidemics following the initial Kenyan outbreak of 2004. Improved modelling parameterisation has been facilitated by rekindled interest in this re-emerging pathogen. Although simple, our model incorporates a biological component of chikungunya infection that appears to have been neglected until now - previous models have not distinguished between infectiousness and the development of symptoms, an important distinction in chikungunya infection [31], [32]. Sensitivity analysis shows the criticality of the rate of symptoms onset, thereby supporting its inclusion in future modelling efforts. Our results demonstrate the necessity of distinguishing the rate of symptoms onset (inverse of the pre-patent period) from the rate at which an individual becomes infectious (inverse of the latent period of infection), both of which are independently influential parameters in our model (Figure 3).

Generating reliable estimates for the basic (and type) reproduction number and providing a thorough sensitivity analysis of model inputs is particularly important during the initial stages of infectious disease epidemiological research. Both sets of metric are critical to informing control. The *R_T_* value is fundamental to assessing the risk of epidemics and discriminates between epidemiological settings with high and low rates of transmission. It also provides a definitive goal for interventions: the reduction of *R_T_* below the epidemic threshold. Control can then be strategized according to the sensitivity of epidemiological outcomes to model inputs. Typically, control consists of vector control. However, there are logistical difficulties with eliminating the widespread and abundant *Aedes* spp. vectors [25], [33], and, in the absence of effective treatment [4] there is a desperate need for more strategic tools in controlling chikungunya. Our analysis demonstrates a strong influence of the rate at which hosts become infectious (inverse of the latent period) to both the peak incidence and total infected population. Therefore, we propose that pre-emptive isolation of recently bitten individuals i.e., suspected infections, can be expected to attenuate the course of an epidemic. In situations such as the Réunion outbreak, where over a quarter of a million individuals were infected, quarantining might become logistically impossible. In which case, region-wide efforts to reduce transmission from infected individuals to mosquitoes (such as with the use of mosquito repellents) should be employed. Our study demonstrates the substantial improvement to control that can result from pre-emptive action, and our methods should be adaptable to other diseases whereby infectiousness precedes the onset of symptoms.
